# Shiga Toxin and Lipopolysaccharide Induce Platelet-Leukocyte Aggregates and Tissue Factor Release, a Thrombotic Mechanism in Hemolytic Uremic Syndrome

**DOI:** 10.1371/journal.pone.0006990

**Published:** 2009-09-11

**Authors:** Anne-lie Ståhl, Lisa Sartz, Anders Nelsson, Zivile D. Békássy, Diana Karpman

**Affiliations:** Department of Pediatrics, Clinical Sciences Lund, Lund University, Lund, Sweden; Charité-Universitätsmedizin Berlin, Germany

## Abstract

**Background:**

Aggregates formed between leukocytes and platelets in the circulation lead to release of tissue factor (TF)–bearing microparticles contributing to a prothrombotic state. As enterohemorrhagic Escherichia coli (EHEC) may cause hemolytic uremic syndrome (HUS), in which microthrombi cause tissue damage, this study investigated whether the interaction between blood cells and EHEC virulence factors Shiga toxin (Stx) and lipopolysaccharide (LPS) led to release of TF.

**Methodology/Principal Findings:**

The interaction between Stx or LPS and blood cells induced platelet-leukocyte aggregate formation and tissue factor (TF) release, as detected by flow cytometry in whole blood. O157LPS was more potent than other LPS serotypes. Aggregates formed mainly between monocytes and platelets and less so between neutrophils and platelets. Stimulated blood cells in complex expressed activation markers, and microparticles were released. Microparticles originated mainly from platelets and monocytes and expressed TF. TF–expressing microparticles, and functional TF in plasma, increased when blood cells were simultaneously exposed to the EHEC virulence factors and high shear stress. Stx and LPS in combination had a more pronounced effect on platelet-monocyte aggregate formation, and TF expression on these aggregates, than each virulence factor alone. Whole blood and plasma from HUS patients (n = 4) were analyzed. All patients had an increase in leukocyte-platelet aggregates, mainly between monocytes and platelets, on which TF was expressed during the acute phase of disease. Patients also exhibited an increase in microparticles, mainly originating from platelets and monocytes, bearing surface-bound TF, and functional TF was detected in their plasma. Blood cell aggregates, microparticles, and TF decreased upon recovery.

**Conclusions/Significance:**

By triggering TF release in the circulation, Stx and LPS can induce a prothrombotic state contributing to the pathogenesis of HUS.

## Introduction

Platelets and leukocytes do not interact with each other in the circulation under normal circumstances. Enhanced platelet-leukocyte interactions occur *in vivo* in vascular disease processes, such as unstable angina, acute myocardial infarction, and stroke [1], in polycythemia vera [2] and diabetes [3] as well as during vascular surgical procedures such as cardiopulmonary bypass [1] and aortic valve surgery [4]. The formation of platelet-leukocyte aggregates plays an important role in the initiation of thrombogenesis and inflammation [5]. Upon platelet activation, P-selectin is released and expressed on the cell surface. Binding of P-selectin to its ligand P-selectin glycoprotein ligand 1 (PSGL-1), constitutively expressed on all leukocytes, mediates the formation of platelet-leukocyte aggregates in the circulation and on damaged vascular surfaces on which platelets and fibrin have been deposited [1]. Leukocytes can roll on activated platelets enabling binding and migration into inflammatory tissues. Binding of platelets to monocytes and neutrophils results in expression of the integrin CD11b/CD18 which enables further aggregate formation by interaction with the GPIb receptor on platelets [6], alternatively with fibrinogen bound to the GPIIb/IIIa receptor [7] expressed on activated platelets. Activated platelets, monocytes and neutrophils release microparticles which express tissue factor (TF) [8], [9], [10].

TF is a transmembrane cell surface glycoprotein that acts as a receptor for coagulation factor VII/VIIa, catalyzes the conversion of factor X to the active form Xa and ultimately leads to thrombin formation. Thrombin converts fibrinogen to fibrin resulting in formation of an insoluble fibrin clot [11]. In addition, thrombin is a potent stimulator of platelet activation [12]. Excessive expression of TF occurs in prothrombotic conditions such as sepsis, endotoxemia, systemic lupus erythematosus, atherosclerosis, Crohn's disease, transplant rejection reactions and hemolytic uremic syndrome (HUS) [13], [14], [15], [16], [17], [18].

HUS develops as a complication of Shiga toxin (Stx)-producing enterohemorrhagic *Escherichia coli* (EHEC) infection of which serotype O157:H7 is the most commonly detected. HUS is characterized by non-immune hemolytic anemia, thrombocytopenia and acute renal failure [19]. HUS is a prothrombotic state in which platelets are activated and fibrinolysis is decreased [20], [21], [22]. The mechanisms by which thrombotic microangiopathy occurs during HUS have not been fully elucidated. Stx may contribute to the process by inducing endothelial cell damage [23] an effect enhanced in the presence of lipopolysaccharide (LPS) [24] which increases Stx binding [25]. Stx promotes platelet deposition on endothelial cells exposed to high shear stress [26]. Endothelial cell injury could, potentially, expose the subendothelium releasing thrombogenic factors, such as fibrinogen and von Willebrand factor, which could trigger platelet aggregation. Furthermore, Stx can interact directly with platelets inducing their activation [27].

In the present study the effects of Stx2 and LPS on platelet-leukocyte aggregate formation and TF expression were investigated in order to identify a prothrombotic mechanism contributing to thrombotic microangiopathy. The in vivo relevance of platelet-leukocyte aggregate formation and TF expression was studied in samples from HUS patients.

## Materials and Methods

### Subjects

Venous blood was obtained from 25 human healthy volunteers (16 women, 9 men) not using any medications. Blood samples were also available from three boys and one girl aged 1–9 years (median 2 years), diagnosed with EHEC-associated HUS and treated at the Department of Pediatrics, Lund University Hospital. Patient samples were taken on the first day after admission, while the children had clinical signs of HUS. HUS was defined as hemolytic anemia (hemoglobin levels <100 g/L), elevated lactic dehydrogenase, thrombocytopenia (platelet counts <140×10^9^/L) and acute renal failure. Blood was also obtained from all four children two to four months after recovery. Laboratory diagnosis of EHEC infection was determined by PCR and serotyping of fecal isolate serotypes [28]. Fecal isolates were positive for Stx2-producing *E. coli* O157:H7 in three cases and in the fourth case for Stx1- and Stx2- producing *E. coli* O98:H21. Samples were also available from 4 pediatric controls, 3 boys and 1 girl, aged 5–11 years (median 6.5 years). These children were seen at the out-patient clinic of the Department of Pediatrics, Lund University Hospital, for follow-up of posterior urethral valve, recurrent urinary tract infections or neuroblastoma. None of the pediatric controls had a history of diarrhea or HUS. Samples were also obtained from three patients with acute renal failure, 2 males aged 25 and 62 years, respectively, and one girl from which blood was obtained at 7 days of age. The adult patients were treated at the Department of Nephrology of the University Hospitals of Malmö and Lund for renal failure due to vitamin D intoxication and acute tubular necrosis, respectively. The child was treated at the Department of Pediatrics, Lund University Hospital for renal failure after heart surgery.

### Ethics statement

Samples from healthy donors, patients and pediatric controls were taken with the informed written consent of the subjects or their parents and with the approval of the Ethics Committee of the Medical Faculty, Lund University.

### Blood collection

Blood was drawn by venipuncture via a butterfly needle (Plasti Medical S.p.A, Villamarzana, Italy) with low tourniquet or through an intravenous cannula (Neoflon™Cannula, Becton Dickinson, Franklin Lakes, NJ). The first 2 mL were discarded and the required volume collected into plastic tubes containing 0.27 mL 0.109 M sodium citrate (Becton Dickinson, Plymouth, UK) or heparin (68 IU, Beckton Dickinson). After blood sampling the blood was diluted (1∶2) in sterile LPS-free RPMI-1640 (Invitrogen, Paisley,UK) containing Gly-Pro-Arg-Pro (GPRP, 10 mM; Sigma-Aldrich, St. Louis, MO), to prevent the polymerization of fibrin molecules. Samples were used as whole blood (sodium citrate) in flow cytometry and flow chamber perfusion experiments or centrifuged at 2000×g for 15 minutes to obtain platelet-poor-plasma used to quantify microparticles or for tissue factor activity assays. Plasma was stored at −80°C until used. Heparinized samples were used for purification of leukocytes as described below.

### Study design

#### Stimulation of whole blood by Stx2 or LPS

Whole blood diluted in RPMI-1640/GPRP was incubated with purified Stx2 (200 pg/mL, final concentration in all experiments, diluted in phosphate-buffered saline (PBS, pH 7.4, Medicago, Uppsala; Sweden)) a gift from T.G. Obrig, Department of Microbiology and Immunology, University of Maryland School of Medicine, Baltimore), LPS (purified by phenol extraction and used at 0.5 µg/mL or 1 µg/mL, final concentration in all experiments defined by the limulus amebocyte lysate assay, LAL, Coatex, Gothenburg, Sweden) from *E. coli* O103:H2, O111:HN, O121:H19 (a gift from P.I. Tarr, Division of Pediatrics, Washington University School of Medicine, St Louis, MO) [29], O157:H7 (a gift from R. Johnson, Public Health Agency of Canada, Guelph, Canada), non-EHEC-LPS O111:B4 (Sigma Aldrich) or a combination of Stx2/LPS or PBS for 4 h at 37°C. The LPS content of the Stx2 preparation was assayed by the LAL assay and found to be less than 50 pg/mL LPS (the detection limit).

### Detection of platelet-leukocyte aggregates

To detect platelet-monocyte aggregates whole blood was incubated with mouse anti-human CD42b:RPE-Cy5 (1∶50, to detect GP1b on platelets) and mouse anti-human CD38:FITC (1∶40, to detect monocytes), simultaneously or mouse IgG_1_:RPE-Cy5 (1∶50) and mouse IgG_1_:FITC (1∶40) as the isotype controls for 10 minutes at room temperature (all antibodies from BD Biosciences, San Diego, CA). Similarly, platelet-neutrophil aggregates were detected by simultaneous incubation with mouse anti-human CD42b:RPE-Cy5 and mouse IgM anti-human CD66:FITC (1∶40, BD Biosciences, to detect neutrophils), or mouse IgG_1_:RPE-Cy5 and mouse IgM:FITC (1∶40, BD Biosciences) as the antibody controls for 10 minutes at room temperature. Erythrocytes were lysed by incubation with FACSLyse (Dako, Glostrup, Denmark). Samples were stored at 4°C and analyzed by flow cytometry within 30 minutes of lysis. Events staining positively for both platelet and monocyte antigens or platelet and neutrophil antigens were considered to represent blood cell aggregates, as assayed by flow cytometry described below.

### Detection of platelet and leukocyte activation

To demonstrate the activation-dependent integrin receptor GPIIb/IIIa on platelets a three-antibody mixture was separately added to each sample of whole blood: mouse anti-human PAC-1:FITC (1∶100, to detect the activated GPIIb/IIIa receptor, Becton Dickinson, San Jose, CA), mouse anti-human CD42b:RPE-Cy5 and mouse anti-human CD38:RPE (1∶60, BD Biosciences) or mouse anti-human CD66:RPE (1∶50, BD Biosciences). The activation-related antigen CD11b on leukocytes was detected by incubation with a mixture of mouse anti-human CD11b:FITC (1∶10, Dako), mouse anti-human CD42b:RPE-Cy5 and mouse anti-human CD38:RPE or mouse anti-human CD66:RPE. Mouse IgG_1_:FITC (1∶10, Dako) and mouse IgM:FITC (1∶100, Becton Dickinson) were used as control antibodies for CD11b and PAC-1, respectively.

### Tissue factor expression

A three antibody mixture was added to whole blood containing mouse anti-human CD142:RPE (1∶30, BD Biosciences) to detect tissue factor, mouse anti-human CD42b:RPE-Cy5 and mouse anti-human CD38:FITC or mouse anti-human CD66:FITC or their respective control antibodies. Mouse IgG_1_:RPE (1∶30, BD Biosciences) was the control for the anti-CD142:RPE antibody.

### Microparticle isolation

Whole blood was stimulated with agonists as described above and centrifuged to platelet-poor-plasma. Microparticles were isolated as previously described [18] and incubated with Annexin V-Cy5 (1∶100, BD Biosciences) [18], mouse anti-human tissue factor CD142:RPE and mouse anti-human CD42b:FITC or mouse anti-human CD38:FITC, alternatively mouse anti-human CD66:FITC, simultaneously or isotype controls IgG_1_:FITC and IgG_1_:PE (all from BD Biosciences).

### Isolation of platelets, neutrophils, and monocytes

Platelets were purified from platelet-rich-plasma as previously described [21]. Neutrophils and monocytes were isolated from heparinized peripheral blood collected from healthy donors using a one-step density gradient centrifugation with Polymorphprep® (Nycomed, Oslo, Norway). Leukocytes were resuspended in RPMI-1640 at a concentration of 1×10^6^/mL.

### Binding of Stx2 and O157LPS to platelets and leukocytes

Binding of Stx2 to platelet-leukocyte aggregates or to free (unbound) platelets, monocytes or neutrophils was assayed in whole blood, incubated with Stx2 (200 pg/mL) for 30 min at 37°C and the antibody combinations described above. Erythrocytes were lysed with FACSLyse and cells were washed twice with PBS and incubated with a mouse monoclonal anti-Stx2 IgG_1_ antibody (11E10, 200 ng/mL, a gift from T.G. Obrig) or mouse IgG_1_ (Dako) diluted in 0.1% Saponin (Sigma-Aldrich)/PBS for 30 min at room temperature. After washing, the blood cells were incubated with rabbit anti-mouse:RPE F(ab′)_2_ (1∶1000, Dako, diluted in 0,1% Saponin/PBS), as the secondary antibody for 30 min at room temperature. Specificity of the secondary antibody was tested by omitting the primary antibody. Stx2 binding was also examined on purified platelets, monocytes and neutrophils and binding was detected in a similar manner. O157LPS binding was detected as previously described [21].

### Flow cytometry acquisition and interpretation of data

**Figure 1:**
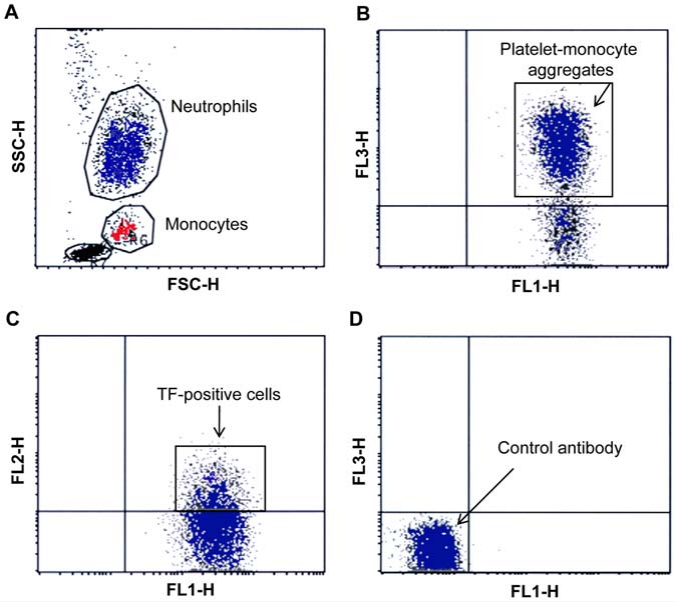
Detection of platelet-monocyte or platelet-neutrophil aggregates and tissue factor expression by flow cytometry. (A) The neutrophil and monocyte population were identified in whole blood by their characteristic size and granularity. In the monocyte gate 98% of the cells were positive for the monocyte marker CD38:FITC and 99% of the cells in the neutrophil gate were CD66:FITC positive showing the accuracy of the identification of cells by forward and side scatter. (B) Monocytes or neutrophils in complex with platelets (gate 2), were identified by binding of CD38:FITC or CD66:FITC (FL1) and the platelet specific antibody CD42b:RPE-Cy5 (FL3). Cells in gate 3 represent platelet-free monocytes or neutrophils. (C) Surface bound tissue factor was identified by binding of CD142:RPE and CD38:FITC or CD66:FITC (FL2 vs. FL1). (D) Percentage of positive cells was calculated by subtraction of the negative control antibody.

Flow cytometry was performed using a FACSCalibur instrument with CELLQuest software (Becton Dickinson Immunocytometry Systems, San Jose, CA). Forward and side scatter measurements were made with gain settings in linear mode for the analysis of platelet-monocyte or platelet-neutrophil interactions. The monocyte and the neutrophil populations were thus easily distinguished (Figure 1A). A minimum of 2000 monocytes and 5000 neutrophils were acquired for each determination. A three-color analysis was used for simultaneous detection of platelet-leukocyte aggregates and platelet-leukocyte activation. Monocytes and neutrophils were further gated into a CD42b-positive platelet-bound population, gate two, and a CD42b-negative platelet-free population, gate three (FL1 versus FL3, Figure 1B).

Determination of surface-bound TF or Stx on platelet-leukocyte aggregates was performed using a three-color analysis procedure for simultaneous detection of platelet-leukocyte aggregates and TF or Stx2 presence on blood cell aggregates. In the platelet-bound leukocyte population (gate two) the FL1 versus FL2 cytograms were used to estimate the percentage of platelet-leukocyte aggregates with bound TF or Stx2 (Figure 1C). Similarly, the FL1 versus FL2 cytograms were used to estimate the percentage of TF or Stx2 in the platelet-free leukocyte population. Unbound platelets were measured with forward and side scatter signals in logarithmic mode. 10 000 events were acquired with a live gate on FSC versus SSC. Results were compared to antibody-matched controls (Figure 1D) and expressed as percentage positive cells or as mean fluorescence intensity (MFI) after subtraction of the control antibodies. Microparticles were defined and detected as previously described [18].

### Flow chamber perfusion studies

Whole blood was perfused through a modification of a parallel flow chamber [30] surrounded by an incubation hood, adjusting the temperature to 37°C. Defined shear rates between 100–340 s^−1^ and 1000–2000 s^-1^ were generated with a syringe pump in withdrawal mode (Harvard Apparatus Inc, Holliston, MA). Immediately before perfusion PBS (1∶500, PBS:blood), Stx2, O157:H7LPS, O111:B4LPS or a combination of Stx2/O157:H7LPS or Stx2/O111:B4LPS were added to the blood. After 15 minutes perfusion the blood was collected and kept on ice for flow cytometry experiments as described above or centrifuged to platelet-poor-plasma for measurement of TF procoagulant activity.

### Tissue factor procoagulant activity assay

Tissue factor procoagulant activity (TF) in plasma was measured with Actichrome® TF (product nr 846, American Diagnostica Inc, Stamford, CT). Whole blood was incubated with Stx2 (200 pg/mL) and/or O157:H7LPS (1 µg/mL) or O111:B4LPS (1 µg/mL) for 4 h before centrifugation to obtain platelet-poor-plasma. Plasma samples (from flow chamber experiments or after 4 h incubation with the agonists) were used. Samples were stored at −80°C until assayed.

### Statistical analysis

Differences between whole blood and isolated blood cells, incubated with the various stimulants or left unstimulated, were assessed by the Mann-Whitney U-test. A *P*-value of 0.05 or lower was considered significant. Statistical analyses were performed using SPSS version 11 (SPSS, Chicago, IL).

## Results

### Stx2 and EHEC-LPS induce platelet-leukocyte aggregate formation

**Figure 2:**
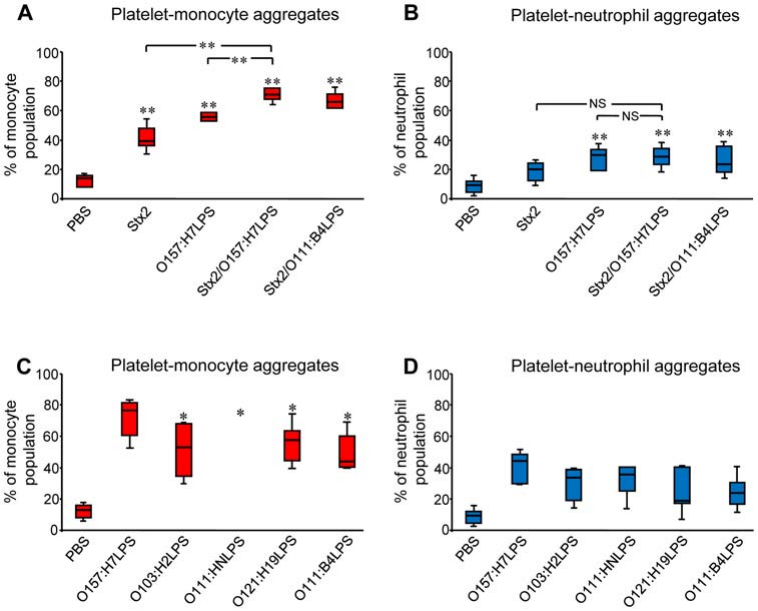
Platelet-monocyte and platelet-neutrophil aggregates induced by incubation with Stx2 and/or LPS determined by flow cytometry. (A) Incubation of PBS-treated whole blood at 37°C for 4 h induced a slight increase in platelet-monocyte (A) and platelet-neutrophil (B) aggregate formation compared to baseline levels. Baseline levels of platelet-monocyte aggregates were 10% (range 5–17%, 7 experiments) and platelet-neutrophil aggregates 6% (range 2–13%, 7 experiments). Incubation of whole blood with Stx2 and/or O157LPS (0.5 µg/mL) induced predominantly the formation of platelet-monocyte aggregates. (C) Incubation of whole blood with O157LPS (1 µg/mL) at 37°C for 4 h induced significantly more aggregate formation between platelets and monocytes in comparison to the other LPS serotypes tested while no significant differences, between LPS serotypes, were observed in platelet-neutrophil (D) aggregate formation. Results are expressed as the percentage of the monocyte or neutrophil population that was positive for CD38:FITC or CD66:FITC as well as the platelet specific marker CD42b:RPE-Cy5. Data are expressed as mean±standard deviation (n = 10 experiments), ** denotes *P*<0.01 when comparing aggregate formation in whole blood incubated with a stimulant and PBS-treated whole blood and * denotes *P*<0.05, comparing aggregate formation in whole blood incubated with O157LPS with those incubated with O103, O111, O121, or O111:B4LPS. NS; indicates not significant.

Incubation of whole blood with Stx2 for 4 h induced a significant increase in platelet-monocyte but not platelet-neutrophil aggregate formation (Figure 2A and 2B) while LPS induced both platelet-monocyte and platelet-neutrophil aggregate formation (Figure 2A and 2B). All LPS serotypes tested (O157:H7, O103:H2, O111:HN, O121:H19 or O111:B4) induced increased aggregate formation, although O157:H7LPS was more potent compared to the other serotypes (Figure 2C and 2D). For both stimulants more aggregates were observed between platelets and monocytes than between platelets and neutrophils.

A significant increase in platelet-monocyte but not platelet-neutrophil aggregate formation was noted when blood cells were co-stimulated with Stx2 and LPS simultaneously, in comparison to Stx2 or LPS alone (Figure 2A and 2B). The results show that both Stx2 and O157LPS induce platelet-monocyte aggregate formation, that co-stimulation with Stx2 and LPS had an additive effect and that O157LPS was a more potent trigger of platelet-leukocyte aggregate formation than other LPS serotypes.

### PAC-1 binding to leukocyte-bound and -free platelets as a marker for platelet activation

**Figure 3:**
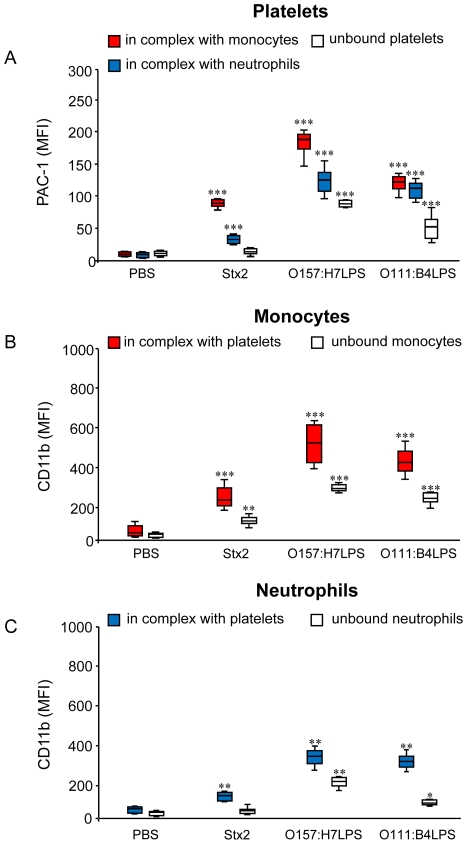
Activation status of platelets, monocytes, and neutrophils stimulated with Stx2, and LPS. (A) Incubation of whole blood with Stx2, O157:H7LPS (1 µg/mL) or O111:B4LPS (1 µg/mL) induced PAC-1 binding to platelets in complex with monocytes or neutrophils as determined by flow cytometry. LPS also induced PAC-1 binding to unbound platelets. (B) Stx2, O157:H7LPS or O111:B4LPS stimulation induced CD11b expression on monocytes and (C) on neutrophils in complex with platelets although the effect of Stx2 on neutrophils was minimal. For all markers O157LPS was more potent than all other LPS serotypes tested. Results are expressed as a mean of mean fluorescence intensity (MFI)±standard deviation (six experiments), *** denotes *P*<0.001, ** *P*<0.01 and * *P*<0.05 when comparing PAC-1 or CD11b expression in whole blood incubated with stimulant with PBS-treated whole blood.

Platelet activation was detected in whole blood after incubation with Stx2, O157LPS or O111:B4LPS by binding of the PAC-1 antibody to platelets in complex with monocytes or with neutrophils (Figure 3A) as well as to unbound platelets stimulated with LPS. An increase in binding of the PAC-1 antibody was detected on leukocyte-bound platelets in whole blood stimulated with Stx2 although no effect was noted on unbound platelets. Whole blood stimulated with LPS exhibited even more platelet activation in comparison to whole blood incubated with Stx2 and the PBS-control.

### Surface expression of CD11b on platelet-bound and platelet-free monocytes and neutrophils as a marker of leukocyte activation

Whole blood incubated with Stx2 exhibited increased monocyte and neutrophil surface expression of CD11b (Figure 3B and 3C), albeit to a lesser extent compared to LPS (O157:H7 or O111:B4). Stx2 stimulation increased CD11b expression in platelet-bound as well as in platelet-free monocytes whereas only a weak increase was detected in neutrophils in complex with platelets and no increase was seen in platelet-free neutrophils (Figure 3C). LPS stimulation increased the intensity of CD11b staining on monocytes and neutrophils in complex with platelets more than visualized on unbound monocytes/neutrophils. Incubation with PBS did not induce increased CD11b surface expression. Thus both Stx2 and O157LPS induced platelet and leukocyte activation which was more notable when cells were in complex.

### Stx2 and EHEC-LPS induce tissue factor expression on platelet-leukocyte aggregates

**Figure 4:**
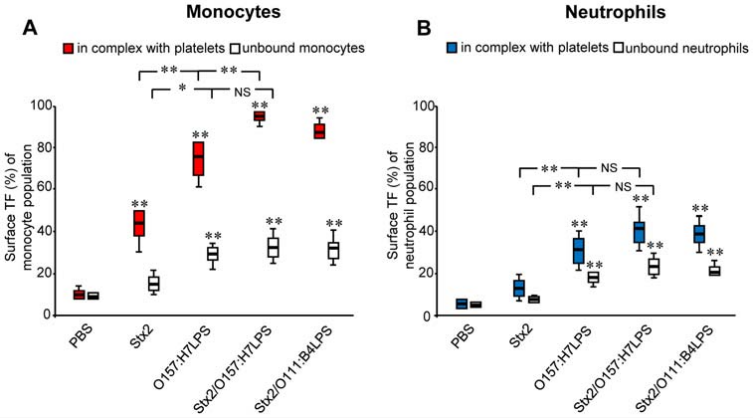
TF expression on blood cells in complex and free cells induced by incubation with Stx2 and/or LPS. (A) Incubation of whole blood with Stx2 or O157:H7LPS (0.5 µg/mL) induced TF expression mostly on platelet-monocyte aggregates and to a lesser degree on unbound monocytes as determined by flow cytometry. (B) Likewise, in the neutrophil population TF expression was mostly seen on neutrophils in complex with platelets. Data are expressed as percentage of the platelet-monocyte or platelet-neutrophil population or percentage of unbound monocytes or neutrophils that were positive for the TF antibody±standard deviation (n = 10). ** denotes *P*<0.01 and * *P*<0.05, when comparing TF expression on aggregates in whole blood incubated with Stx2, LPS or the Stx2/O157LPS combination with unstimulated PBS-treated whole blood (except when comparisons are delineated). NS; indicates not significant.

**Figure 5:**
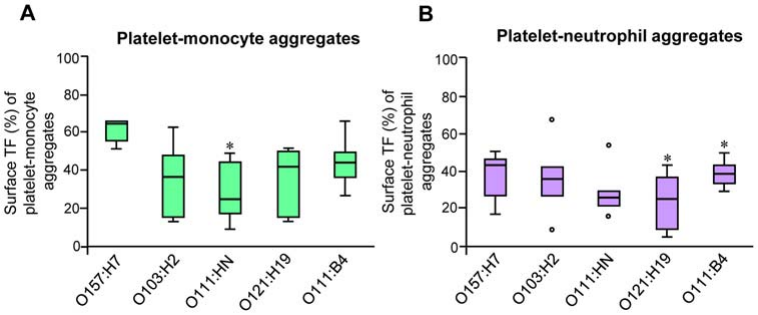
LPS–induction of TF expression on platelet-leukocyte aggregates. Incubation of whole blood with O157LPS (1 µg/mL) induced slightly more TF expression compared to other LPS serotypes tested. Data are expressed as percentage of the platelet-monocyte (A) or platelet-neutrophil population (B) that were positive for the TF antibody±standard deviation (n = 10). * denotes *P*<0.05, comparing TF expression on blood cell aggregates incubated with O157LPS with those incubated with O103, O111, O121, or O111:B4LPS.

Directly after blood sampling unstimulated cells in whole blood showed low baseline levels of TF. TF expression was detected on 8% of platelet-monocyte aggregates (range 4–12%, 7 experiments) but not on platelet-neutrophil aggregates or unbound cells (data not shown). Incubation of PBS-treated whole blood at 37°C for 4 h increased the population of platelet-monocyte and platelet-neutrophil aggregates with surface-bound TF minimally (Figure 4A). Incubation of whole blood with Stx2 and/or LPS for 4 h induced a significant increase of platelet-monocyte aggregates expressing TF (Figure 4A) compared to PBS-treated samples. LPS induced increased platelet-neutrophil aggregates with surface-bound TF, whereas Stx only had a negligible effect (Figure 4B). TF bound to monocytes and neutrophils in complex with platelets to a greater extent than to platelet-free leukocytes. Incubation of whole blood with a combination of Stx2 and LPS (O157:H7LPS or O111:B4LPS) induced a significant increase in platelet-monocyte aggregates as well as platelet-free monocytes expressing TF compared to Stx2 or LPS alone. A similar, albeit weaker, effect was noted on platelet-bound or platelet-free neutrophils. All LPS serotypes tested showed similar results although O157:H7LPS was slightly more potent (Figure 5A and 5B).

Taken together these results show that Stx2, O157LPS and Stx2/LPS induce a significant increase in TF expression, predominantly on platelet-monocyte aggregates, that the combination of Stx2 and LPS had an additive effect on TF-expression on platelet-monocyte aggregates, and that O157LPS was slightly more potent than the other LPS serotypes.

### Stx2 and EHEC-LPS induce tissue factor–positive microparticles

Levels of microparticles generated by incubation of whole blood with Stx2 or LPS were significantly higher compared to PBS-treated blood (Table 1). The highest numbers of microparticles were generated by incubation with LPS. O157:H7LPS generated more microparticles than O111:B4LPS. A significant increase in platelet microparticle generation and numbers of microparticles expressing TF was noted in whole blood co-stimulated with Stx2/LPS compared to each agonist alone (*P*<0.05). For all agonists tested platelet microparticles constituted the largest proportion of total microparticles followed by microparticles of monocyte origin. Similarly, TF-positive microparticles were considerably more elevated in whole blood incubated with an agonist than in PBS-treated blood.

**Table 1:** Numbers and cellular origin of microparticles released from blood cells as determined by flow cytometry.

	Annexin V positive microparticles (x10^3^/mL)	CD42b positive microparticles (x10^3^/mL)	CD38 positive microparticles (x10^3^/mL)	CD66 positive microparticles (x10^3^/mL)	TF positive microparticles (x10^3^/mL)
**Agonist**					
**Unstimulated**	280 (249–304)	202 (174–234)	39 (37–62)	0.5 (0.2–0.7)	67 (34–88)
**Stx2**	1958 (1871–2039)*	1088 (821–1298)*	26 (15–34)*	0.4 (0.1–0.6) *	670 (463–847)*
**O157:H7LPS**	3095 (2678–3484)*	2200 (1514–2590)*	189 (132–291)*	2.0 (1.3–2.6) *	1791 (1207–2257)*
**O111:B4LPS**	2314 (2002–2861)*	1564 (1073–1991)*	112 (71–150)*	2.1 (1.5–2.6) *	1195 (813–1385)*
**Stx2/O157:H7LPS**	3484 (2739–3587)*	2901 (2364–3112)*	197 (121–207)*	2.7 (1.7–3.9) *	2816 (2241–3283)*
**Stx2/O111:B4LPS**	2977 (1895–4059)*	2674 (1341–3533)*	188 (58–303)*	2.4 (1.9–3.7) *	1953 (1679–2968)*

Data are expressed as median and range of microparticles positive for each surface marker/mL of plasma from four different experiments. *; denotes *P value* <0.05 comparing microparticle generation in whole blood stimulated with Stx2, O157:H7LPS (1 µg/mL), O111:B4LPS or Stx2/LPS with unstimulated whole blood. The number of microparticles per mL plasma was calculated as previously described [18].

### Stx2 binds to monocytes and to activated platelets

Results shown above indicated that Stx2 alone did not have as profound effect on platelet-leukocyte aggregate formation, platelet or leukocyte activation and TF-positive microparticle generation as LPS, and since this response was mostly demonstrated in monocytes, we examined the ability of Stx2 to bind to platelets, monocytes and neutrophils. Stx2 was added to whole blood or to isolated platelets, monocytes or neutrophils. The results are summarized in Table 2 and showed that Stx bound mostly to monocytes and platelets, particularly after activation, and that minimal binding to platelet-free neutrophils was detected. There was no binding of Stx2 to platelet-bound neutrophils and no binding was detected when the primary anti-Stx2 antibody was omitted.

**Table 2:** Binding of Stx2 to blood cells in whole blood and to isolated blood cells.

		Whole blood	Isolated blood cells	Isolated blood cells fMLP stimulation	Isolated blood cells ADP stimulation
**Unstimulated**		<0.4	<0.4	<0.4	<0.4
**Monocytes**	Platelet-bound	27% (10–55%)**			
	Platelet-free	41% (24–56%)**			
			40% (30–51%)**	65% (58–88%)**	NA
**Neutrophils**	Platelet-bound	<0.4			
	Platelet-free	4% (0.75–7%)**			
			<0.4	<0.4	NA
**Platelets**		8% (5–9%)**	<0.4	NA	49% (36–69%)**

Data are expressed as (%) of cells positive for Stx2 and shown as median and range from five different experiments. NA; not analyzed. **; denotes *P value* <0.01 when comparing Stx2 binding to monocytes, neutrophils or platelets to unstimulated whole blood or to isolated unstimulated cells.

### Stx2 and EHEC-LPS induce platelet-monocyte aggregate formation and tissue factor expression under shear stress

**Figure 6:**
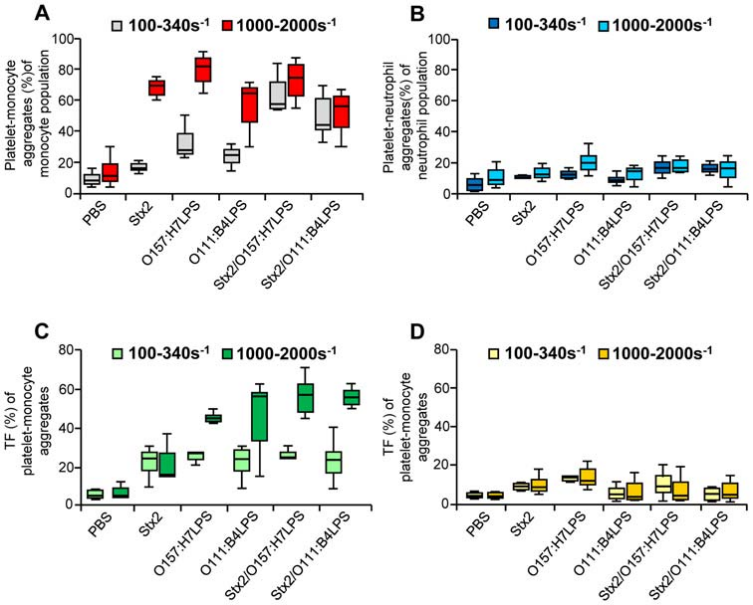
Platelet-monocyte and platelet-neutrophil aggregate formation and TF expression induced by shear and Stx2 and/or LPS. Stx2, O157:H7LPS, O111:B4LPS (both LPS serogroups at 1 µg/mL) or a combination of Stx2 and LPS were added to whole blood immediately before perfusion of whole blood through a flow chamber system and aggregate formation and tissue factor expression determined by flow cytometry. (A) Stx2 and/or LPS induced platelet-monocyte aggregate formation particularly at shear rates between 1000–2000 s^−1^ (light bars). (B) Aggregate formation between platelets and neutrophils increased minimally by addition of Stx2 or LPS at both low (100–340 s^−1^) and high shear rates as compared to PBS-treated samples. (C) Stx2 or LPS induced TF expression on platelet-monocyte aggregates at low shear rates which increased even more at high shear rates. Expression increased markedly in Stx2/LPS stimulated samples at higher shear rates. (D) Stx2 and/or LPS induced only a weak increase in TF expression on platelet-neutrophil aggregates. Higher shear rates did not induce more TF expression. Data are expressed as mean±standard deviation (n = 3). Statistical comparisons were not carried out as only three experiments were performed due to the large amounts of blood required.

Perfusion of whole blood at shear rates between 100–340 s^−1^ or 1000–2000 s^−1^ with Stx2, O157LPS (1 µg/mL) or O157LPS/Stx2 through a parallel plate flow chamber increased platelet-monocyte aggregates considerably (Figure 6A) compared to PBS-stimulated whole blood. Similarly, O111:B4LPS alone or in combination with Stx2 increased platelet-monocyte aggregates. Higher shear rates (1000–2000 s^−1^) induced more platelet-monocyte aggregate formation in the presence of Stx2 or O157LPS than lower shear rates (Figure 6A). Notably, the combination of Stx2/O157LPS at the lower shear rate increased platelet-monocyte aggregates two-fold in comparison to each agonist alone. Combination of low or high shear and Stx2, O157LPS or both simultaneously induced only a slight increase in platelet-neutrophil aggregates (Figure 6B).

Stx2, O157LPS or O111:B4LPS or Stx2/LPS increased the number of platelet-monocyte aggregates with surface-bound TF at both low and high shear rates (Figure 6C). A marked increase in platelet-monocyte aggregates with surface-bound TF was noted in whole blood co-stimulated with Stx2/LPS at higher shear rates. The effect of shear-stress on platelet-neutrophil aggregates expressing TF was minimal (Figure 6D). Thus a most potent additive effect regarding TF-expression was noted on platelet-monocyte aggregates exposed to Stx2/LPS at high shear rates.

### Stx 2 and EHEC-LPS induce release of functional tissue factor into plasma under shear stress

To examine the levels of functional TF in plasma the procoagulant activity of TF was measured. Plasma levels of functional TF were examined after incubation of whole blood with Stx2, O157:H7LPS or O111:B4LPS or LPS/Stx2 for 4 h. All agonists tested, alone or in combination, induced increased plasma levels of functional TF, with a minimal additive effect when agonists were combined (Table 3).

**Table 3:** Release of functional tissue factor into plasma, as determined using ELISA.

	Tissue factor in plasma (pg/mL)	Tissue factor in plasma (pg/mL)	Tissue factor in plasma (pg/mL)
**Agonist**	Incubation at 37°C, 4 h	Shear rate, (s^−1^), 37°, 100–340	Shear rate, (s^−1^), 37°, 1000–2000
**Unstimulated**	60 (52–87)	98 (70–120)	106 (90–130)
**Stx2**	98 (72–112)	163 (127–190)	274 (256–298)
**O157:H7LPS**	110 (93–127)	175 (130–230)	332 (293–377)
**O111:B4LPS**	112 (89–127)	151 (94–176)	276 (241–309)
**Stx2/O157:H7LPS**	125 (97–145)	196 (158–207)	341 (326–367)
**Stx2/O111:B4LPS**	119 (93–135)	181 (148–200)	321 (294–344)

Data are expressed as pg of active tissue factor/mL of plasma and shown as median and range from three different experiments. Whole blood was incubated with agonists for 4 h or exposed to a flow system under controlled shear rates.

Stx2, LPS (O157:H7 or O111:B4) or Stx2/LPS were added to whole blood immediately before perfusion through a parallel plate flow chamber. At low shear rates (100–340 s^−1^) Stx2, both LPS serotypes tested and Stx2/LPS induced increased release of functional TF into plasma in comparison to unstimulated- (Table 3) or PBS- treated whole blood (data not shown). The highest amount of functional TF in plasma was noted at shear rates between 1000–2000 s^−1^ but no additive effect was demonstrated when agonists were combined. Thus both Stx2 and O157LPS induced a marked release of functional TF into plasma, especially when shear forces were added.

### HUS patients have TF–positive circulating platelet-monocyte and platelet-neutrophil aggregates

**Figure 7:**
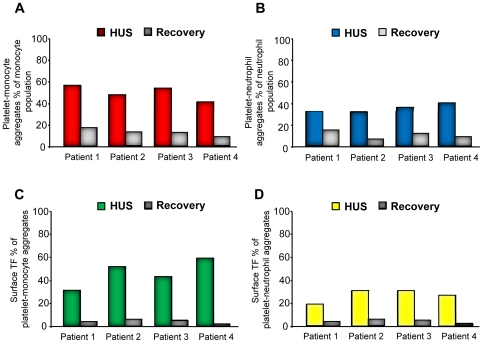
Circulating platelet-leukocyte aggregates and TF expression in patients with HUS. (A) Patients with HUS had increased circulating levels of platelet-monocyte and (B) platelet-neutrophil aggregates during the acute phase of disease compared to levels obtained after recovery. TF expression on platelet-monocyte (C) and platelet-neutrophil (D) aggregates were increased during the acute phase of HUS and decreased at recovery. Samples were available from four patients, thus precluding statistical comparisons.

Platelet-monocyte and platelet-neutrophil aggregates were examined in whole blood from four children with HUS during the acute phase of disease and after recovery. Significantly higher levels of platelet-monocyte as well as platelet-neutrophil aggregates (Figure 7A and 7B) were observed in the patients during the acute phase but not after recovery. Levels at recovery were slightly higher compared to those found in normal adults as shown in Figure 1 (n = 10).

Expression of surface-bound TF was examined during the acute phase of HUS and after recovery. In the acute phase all patients had significantly elevated levels of platelet-monocyte as well as platelet-neutrophil aggregates expressing surface-bound TF as compared to after recovery (Figure 7C and 7D). Levels at recovery were similar to those found in normal adults as shown in Figure 2 (n = 10).

### Plasma from HUS patients has increased levels of TF and TF–expressing microparticles

Plasma levels of functional TF were studied during the acute phase and compared to pediatric controls (n = 4). Plasma TF levels in HUS patients (median 362, range 270–420 pg/mL) were considerably higher than those in pediatric controls (median 102, range 80–120 pg/mL). Plasma from patients during the acute phase of disease showed considerably higher levels of microparticles compared to after recovery (Table 4), most of the microparticles were of platelet origin. TF-expressing microparticles were elevated during the acute phase of disease.

**Table 4:** Numbers and cellular origin of circulating microparticles and TF–expressing microparticles in plasma from HUS patients.

	Annexin V positive microparticles (x10^3^/mL)	CD42b positive microparticles (x10^3^/mL)	CD38 positive microparticles (x10^3^/mL)	CD66 positive microparticles (x10^3^/mL)	TF positive microparticles (x10^3^/mL)
**HUS acute phase**	1568 (1327–2001)	1277 (978–1615)	102 (88–123)	1.9 (1.4–2.3)	782 (593–1216)
**Recovery**	512 (377–619)	407 (335–459)	51 (33–65)	0.6 (0.3–1.0)	163 (92–207)

Numbers and cellular origin of microparticles and microparticles with surface-bound tissue factor/mL of plasma shown as median and range values.

### Stx2 is present on platelet-monocyte and platelet-neutrophil aggregates from patients with HUS

Stx2 was detected on circulating platelet-monocyte (median 39%, range 22–49%) and platelet-neutrophil aggregates (median 24%, range 9–43%) from all patients with HUS during the acute phase of disease. In addition, all patients had Stx2 bound to their platelet-free monocytes (median 37%, range 30–41%), platelet-free neutrophils (median 22, range 17–32%) as well as to their unbound-platelets (median 55%, range 39–82%). After recovery no binding of the antibodies was detected. As a control, no binding of the anti-stx2 antibody was detected on blood cells from the three patients with acute renal failure indicating that antibody binding was not due to cell changes related to acute renal failure.

### O157:H7LPS is present on platelets, monocytes, and neutrophils from patients with HUS

O157:H7LPS was detected on platelets (median 51%, range 35–56%), monocytes (median 55%, range 51–90%) and neutrophils (median 61%, range 49–72%) from three of the HUS patients. Binding of the anti-O157 antibody was not detected on blood cells from the patient from whom the fecal *E. coli* O98:H21 serotype was isolated. Binding of O157:H7LPS to platelet-monocyte or platelet-neutrophil aggregates was not analyzed. After recovery no binding of the antibody was detected.

## Discussion

Tissue factor expressing microparticles are procoagulant [31]. In this study we demonstrate circulating TF-expressing platelet-leukocyte aggregates and microparticles in blood samples from HUS patients. Platelet-leukocyte aggregate formation was generated *in vitro* in normal whole blood stimulated with Stx2 and/or O157LPS under shear flow, microparticles were released and TF was demonstrated on platelet or monocyte-derived microparticles after stimulation. EHEC-associated HUS is known to be a prothrombotic condition [32]. This activity is related to Stx and LPS-induced endothelial cell injury [24] as well as a direct interaction between EHEC virulence factors, Stx and LPS, and platelets, leading to platelet activation [21], [27]. Direct contact between EHEC virulence factors and blood cells, as shown here, leads to TF expression, which would thus trigger thrombosis.

Increased levels of circulating platelet-leukocyte aggregates expressing TF have been found in patients with acute coronary syndrome [33] and in an in vivo model of experimental endotoxemia [34]. TF has been found to play a key role in sepsis [13] and infectious endocarditis [35] and procoagulant microparticles expressing TF have been demonstrated in meningococcal sepsis [36] and in thrombotic microangiopathies [18], [37]. This pro-thrombotic pathway, ultimately generating thrombin, is operational during HUS as it has been shown that TF is elevated during HUS [17]. In vitro studies have shown that TF expression is increased in human glomerular endothelial cells stimulated with Stx1 and TNF-α and further enhanced by exogenous angiotensin II [38]. Similarly, stimulation of macrophage-like THP-1 cells with Stx1 induces expression of TF [39]. Intravenous injection of C57BL/6 mice with Stx2 and/or LPS has been shown to induce TF expression in the kidneys and increase thrombin generation (detected by levels of thrombin-antithrombin III complex) indicating increased coagulability [40]. The mechanism by which TF elevation in the circulation occurs in HUS has not been previously studied. We therefore added EHEC virulence factors to normal whole blood with and without application of shear stress under flow, in order to study their effect on microparticle generation and TF expression. Both Stx2 and LPS induced platelet-leukocyte aggregate formation, TF expression and microparticle generation. Co-stimulation with Stx2 and LPS resulted in an additive effect with regard to platelet-monocyte aggregate formation and TF expression on these aggregates, particularly when high shear forces were applied. The additive effect of both Stx2 and LPS was also demonstrated regarding microparticle generation, especially platelet-derived microparticles. The physiological relevance of these in vitro findings was confirmed in patient samples in which platelet-leukocyte aggregates and TF-bearing microparticles were demonstrated.

Stx has been previously shown to circulate in HUS patients bound to platelets [21] and neutrophils [41], [42] although the binding of Stx on neutrophils has been questioned [43]. We show that Stx did not bind to resting platelets in vitro (Table 2) but bound to activated platelets. This result confirms previous findings [27], [44]. Stx could also bind to monocytes [45] (resting or activated) but did not bind to neutrophils (resting or activated). Interestingly, experiments using whole blood from HUS patients showed that Stx was bound to leukocyte-platelet aggregates composed either of neutrophils or of monocytes but was also present on neutrophils and monocytes that were not in complex with platelets. The discrepancy between our in vitro results and the results from patient blood samples may be explained by other activation factors present in HUS plasma not accounted for in the in vitro experiments. Thus neutrophils circulating during the acute phase of HUS appear to be able to bind Stx and our results indicate that Stx circulates bound to leukocytes, platelets and blood cell aggregates.

High peripheral blood neutrophil counts at presentation of HUS were previously associated with poor outcome [46]. Neutrophil influx into the renal cortex during HUS has been related to severe prognosis and death [47]. Upon activation, neutrophils may express TF [10], tether to and roll on altered endothelium. Higher neutrophil counts may form more aggregates with platelets leading to more TF release, which may, in part, explain why neutrophilia is associated with a worse prognosis.

O157LPS was a more potent inducer of leukocyte-platelet aggregate formation and TF expression than other LPS serotypes. Previous in vitro studies have shown that LPS can induce leukocyte-platelet aggregate formation and TF expression on leukocytes [48], [49] as well as endothelial cells [50]. Furthermore, TF is elevated during systemic endotoxemia [51]. HUS differs from systemic endotoxemia in that there is no consumption of plasma coagulation factors. O157LPS injected intraperitoneally into mice leads to TLR4-mediated thrombocytopenia [21]. The LPS found in the circulation in EHEC-associated HUS is, however, not free in the circulation [52] but bound to patient platelets [21] as well as neutrophils and monocytes, as shown here. The process by which LPS activates platelets and leukocytes, leading to shedding of TF-bearing microparticles, is enhanced in the presence of high shear, which mimics the high shear forces found in vivo in the glomerular capillaries of the renal cortex. This could explain the formation of microthrombi in this location, where the typical lesions is seen in HUS [19].

LPS binds to platelets through the TLR4/P-selectin complex receptor [21] leading to platelet activation, demonstrated by release of CD40 ligand and P-selectin [21]. P-selectin and CD40 ligand have been shown to be potent mediators of inflammation [53], [54]. Expression of these, and other adhesive ligands, triggers platelet-endothelium and platelet-leukocyte interactions. Platelet activation enables the release of chemoattractants and cytokines, which alter the properties of vascular endothelium, promote recruitment of monocytes and induce synthesis of TF by both platelets and monocytes [53]. Although not addressed in this study, endothelial cell damage will also release TF-rich microparticles [53] as could be expected during Stx-induced endothelial injury. In addition, stimulated platelets shed TF-bearing microparticles from their surface [18], [31] and binding of P- selectin on platelets to PSGL-1 on leukocytes will potentiate the generation and shedding of leukocyte-derived microparticles which also bear TF [53]. Thus multiple sources of microparticle generation and TF expression and release may be active during HUS.

### Conclusion

EHEC virulence factors Stx and LPS induce the formation of aggregates between platelets and leukocytes. The effect was additive when whole blood was co-stimulated with both virulence factors and increased under conditions of high shear stress. This process promoted the release of TF-bearing microparticles from activated blood cells. Based on the findings presented in this work we envisage a scenario in which EHEC virulence factors entering the circulation activate platelets and leukocytes. The interaction between these virulence factors and blood cells under shear flow allows blood cell aggregate formation and decryption of TF [48], expression of active TF on cell membranes as well as secretion of TF-rich microparticles. This process will be enhanced by circulating cytokines [55] and by Stx-induced vascular injury releasing endothelial-derived microparticles. Thus microparticles expressing TF will promote thrombogenesis during HUS.
